# Modeling and verification of authentication threats mitigation in aspect-oriented mal sequence woven model

**DOI:** 10.1371/journal.pone.0270702

**Published:** 2022-07-06

**Authors:** Ubaid Ullah, Rizwan Bin Faiz, Muhammad Haleem

**Affiliations:** 1 Department of Computer Science and Information Technology, BRAINS Institute, Peshawar, Pakistan; 2 Faculty of Computing, Riphah International University, Islamabad, Pakistan; 3 Department of Computer Science, Kardan University, Parwan-e-Du Square, Kabul, Afghanistan; University College of Engineering Tindivanam, INDIA

## Abstract

The modeling of security threats is equally important as the modeling of functional requirements at the design stage of software engineering. However, unlike functional requirements modeling, the modeling of security threats is neglected, which consequently introduces software defects during the early stages of software engineering. Hence, there is a need to mitigate these threats at the design stage. Security threats, specifically authentication threats, crosscut other functional and non-functional requirements when modeled using the object-oriented paradigm. This not only makes the design complex but also results in tangling and scattering problems. We therefore model authentication threats using the aspect-oriented modeling (AOM) technique since it separates crosscutting concerns and localizes them as separate units called aspects. Our main research aim is to remove scattering and tangling in security threats modeling using all the core features of the aspect-oriented technique. In this paper, we propose a research approach to model security threats and their mitigation in mal sequence diagram. Using this approach, our contribution makes a clear difference from previous work. Our first contribution is the modeling of authentication threats in the mal sequence diagram using the security profile and AOM profile. Our second contribution is the mathematical verification of the aspect-oriented mal sequence woven model in terms of correctness and completeness. Using the proposed approach, the scattering and tangling from the resultant woven model are successfully removed at the design stage. Thus, the complexity of models and the time and effort required for future modifications of design models are reduced.

## Introduction

**Fig 1:**
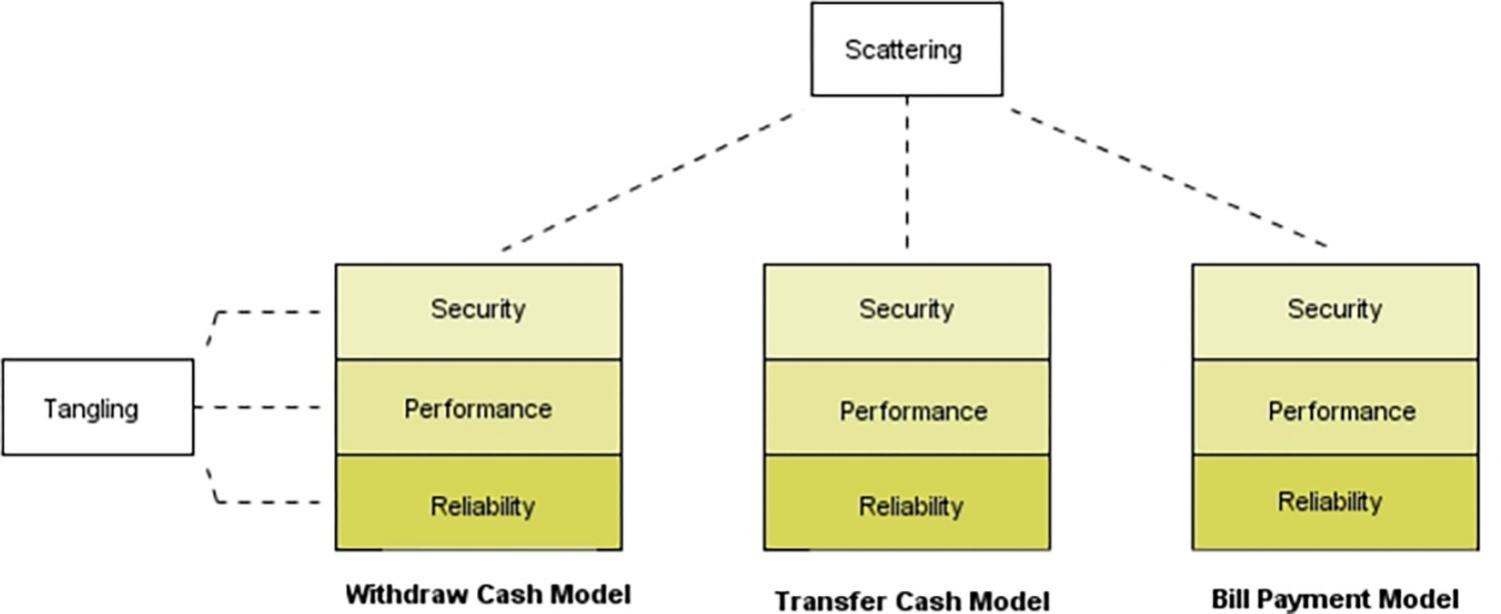
Scattering and tangling.

Modeling the functional and non-functional requirements of software is extremely essential to facilitate successive development activities. The software professionals also focused on modeling functional aspects of software but mostly ignored behavior modeling of non-functional requirements (NFRs) [1,2], and as a result, the research on these NFRs is frequently overlooked [3,4]. Among these NFRs, the most important NFR is security [5–8], which should be planned earlier in the design phase [9]. Security has made significant contributions to software to model and prevent severe threats [10,11]. Among the security attributes, authentication is the most important attribute that relates to the assurance and validation of a user’s identity [12]. Users are first validated to access information stored on a network or computer. When logging into a network, a user must provide unique log-in credentials, such as a user name and password, to protect the network from hacker infiltration. Authentication requirements, from a designer’s perspective, pose security threats that must be addressed. Moreover, various strategies must be considered properly when the addition of authentication requirements is taking place in the later phase of the software lifecycle [13]. This is because the addition of the authentication mechanism to the software in the later phase, i.e., the maintenance phase, further threatens the core functionalities and produces additional defects. These defects [14] also result in financial loss [15]. Therefore, if we try to plan authentication at the design phase, the overall software development budget along with the defects [16] that are injected at this phase [17,18] can be reduced. Furthermore, security behavior is a crosscutting concern [19–21] that affects software core concerns [22] and cannot be separated easily from software functional models through object-oriented techniques. These security concerns produce scattering and tangling issues [23,24] in software models. Scattering occurs when a security concern is spread across different software models, while tangling occurs when a single model hosts multiple different concerns. Both the problems are shown in Fig 1.

These problems result in software models that are difficult to maintain and are highly complex. Therefore, it is not useful for systems like critical systems to model security behavior in this manner [19]. To overcome these problems, the aspect-oriented modeling (AOM) [25] technique is used. The AOM technique separates the concerns [26] that crosscut other design concerns of the software and modularize them. Another challenge for security modeling is that the unified modeling language (UML) allows for the modeling of functional requirements but it does not provide semantics to facilitate the modeling of quality requirements such as security. To solve this problem, the UML provides an extension mechanism to extend the metamodel for software security. Using this mechanism, the security profile and AOM profile are created by extending the metamodel [27]. Moreover, the new extended elements must follow the semantics of UML elements [28]. To add important security constraints to the models, a widely known constraint language, OCL is also extended using the same extension mechanism.

Lastly, we require a UML class and a sequence diagram to implement the research approach. We use the sequence diagram because it shows the dynamic behavior and basic flow of several objects within a single use case. The sequence diagram is good for visualizing interactions among different objects in the class diagram. Software developers use this diagram to document and understand the software [29]. Furthermore, the sequence diagram is useful in model-based testing for checking the functionality. In the proposed approach, the security is modeled through the mal class diagram and mal sequence diagram. The security profile is used for both mal diagrams, while the AOM profile is mainly used for the mal sequence diagram. The ATM case study is used for validation. The verification of the output model is done through mathematical theorems in terms of correctness and completeness.

The structure of the paper is as follows. Section 2 discusses the prior work related to modeling the security attributes and other non-functional attributes. Section 3 discusses the proposed methodology and the modeling steps. Section 4 is about the UML profiling, object constraint language, and AOM features. Section 5 is about the implementation of the ATM case study. Section 6 is related to the verification of the output model. Section 7 is related to the significance of the research, and Section 8 is about the research conclusion.

## Related work

In the following sections, we present important research contributions relevant to AOM. Related work is divided into three different contributions. The first contribution is related to the modeling of security and other quality attributes, the second contribution is related to general approaches used for modeling in AOM and the third contribution is related to the authentication mechanism proposed for the Internet of things (IoT), cyber-physical systems and networks.

### Quality attributes modeling

Ray et al. [30] addressed crosscutting access control concerns in class and collaboration diagrams using an AOM approach. Using the weaving feature, the access control aspect is weaved into the base design to form an application design. The aspect is used as a general design for various applications that require a similar level of access control requirements. This contribution, however, has several limitations. The authors ignored using key AOM features such as pointcut, join point, and advice. No UML extension mechanism is found in the paper. No verification method is used for the proposed work. Mouheb et al. [25] used a systematic generic AOM approach for modeling the security attributes. All the AOM concepts are used in this approach. The approach supports the class diagram and all UML behavioral diagrams. However, no UML extension mechanism is found in their proposed work. Georg et al. [31] proposed the AOM technique to model highly secure software systems. Class and sequence diagrams are used for this purpose. The authors performed a risk assessment to find the potential threat to the software system. The security-driven model is created by integrating the authentication and authorization mechanisms with the core functional model. The resultant woven model is examined to check whether the security-driven model is resistant to the specific attack. However, no threat-oriented UML extension profile is found in their proposed work. The authors also did not use the core AOM concepts like pointcut, join point, and advice. Xu et al. [32] concentrated on the modeling of software functions and their integrity threats using Petri-nets. Petri nets-based aspects are modeled for the prevention of integrity threats to secure the software systems. These aspects are weaved into the functional model to form a software security design. However, no extension profile for security modeling is proposed. Cooper et al. [33] proposed an AOM approach to provide a repository for aspects. These aspects are depicted using a class diagram. The approach, also known as FDAF, uses AOM concepts like aspect, joint point, and advice. Using the AOM weaving feature, the authentication aspect is weaved into a functional design. However, this research is quite simple, and the authors did not employ the UML extension for security. There is no software tool employed for modeling, and no verification of the proposed work is found. The AOM core feature, i.e. pointcut, is missing in the proposed work.

Although security threats modeling is evident from the literature, other non-functional attributes such as reliability, robustness, context-awareness, etc. are also being modeled. However, in these papers, no aspect-oriented extension mechanism for UML models is found, and no verification of the woven model has been done.

Shaukat et al. [1] presented a methodology to support the modeling of crosscutting aspects, i.e., robustness behavior in UML state machines. The video conference system is taken as a case study for validation purposes. Qiu and Zhang [34] proposed an AOM modeling approach to specify the crosscutting reliability tactics as localized aspects using a component diagram. These reliability tactics are weaved into the functional model using a weaving mechanism. The authors [35] proposed the AOM method for cloud computing. This method is used to address the concerns, i.e., running time, reliability, and failure processing. Weaving the schemas, i.e., the base layer, a meta-layer, and meta-object protocol into the main resource model is done dynamically by a weaving mechanism to form a resource scheduling model. Lidia [36] presented a process to address the problems of context-aware systems. The aspect-oriented technique is used to encapsulate the context-awareness concerns as aspects. The key reasons for using the AOM for these systems are to reduce complexity, redundancy, maintenance, correctness, and reusability.

### AOM general approaches

Further literature is related to general approaches used for modeling in AOM. The authors [37,38] proposed an AOM approach for complex systems that are based on the reusability of aspects. The approach supports the modeling of the static structure and dynamic behavior of a system as aspect models. Kompose [39] and Geko [40], are used to implement the weaver in the tool for behavioral and structural diagrams, while the aspect optima is used as a case study [41,42] to implement the approach. Kiczales [43] proposed a mechanism for the extension of the AOM concept, i.e., join point in various UML diagrams. In the paper [44], the authors discussed an AOM approach to determine the crosscutting requirements and consider them as aspects. These aspects are further mapped to the design. Further research in AOM [45–49] reveals that some core constructs of AOM are extended using UML extensions, and these extensions are mostly related to programming.

### Authentication-based techniques

In the paper [50], tewari and gupta proposed a mutual authentication technique between an IoT device and a server based on elliptic curve cryptography (ECC) to deal with attack resistance and communication overhead; whereas, in the paper [51], these authors discussed the vulnerability faced by the ultra-lightweight protocol and proposed a new protocol to tackle the issues. The timestamp is used in the new protocol to improve security against disclosure and de-synchronization. Mirsadeghi et al. [52] suggested an authentication approach for clustered vehicle ad hoc networks that minimized time and cost while detecting rogue nodes. The suggested technique’s major goal is to establish trustworthy and stable clusters that lead to the overall network’s stability. Gupta et al. [53] presented an authentication technique to validate the user before accessing medical data stored on the server. The authentication method also makes use of machine learning and smart card blocking mechanisms. The authentication is tested against various attacks. Nguyen et al. [54] suggested a cyber-physical system intrusion detection system for the health care industry to safeguard data; blockchain technology is also employed for secure data transfer to the cloud server. Gupta et al. [55] proposed a cloud-based cyber-physical system for the online storage and retrieval of personal health data. The proposed system also uses blockchain technology, which has two benefits. First, the system must be free of a single point of failure. Second, it minimizes the need for trusted authority and minimizes the load on data consumers. Gaurav et al. [56] emphasized the need to establish appropriate security measures to strengthen the resilience of cloud-based medical internet of things (MIoTs) to attacks. Furthermore, the authors examined the key security and privacy challenges related to IoMT and provided an overview of the existing approaches. Lu et al. [57] suggested an edge-assisted authentication approach in the cyber-physical system that aims to safeguard the system against unwanted access while reducing the workload for resource-constrained components. Audithan et al. [58] proposed an efficient authentication system for distributed mobile cloud computing. This system supports mutual authentication to protect against fraudulent mobile users and service providers by adopting a third-party revocation mechanism. Vijayakumar et al. [59] designed a trusted authority (TA) for users with a range of online premium services via VANETs. To effectively prevent illegal cars from joining the VANET, the authors proposed a dual authentication approach that provides a high level of security on the vehicle side. Khan et al. [60] offered a strong anonymous authentication technique based on biometrics for digital rights management systems. The suggested technique is verified for correctness. The scheme’s performance is also evaluated using computation and communication time. Zhou et al. [61] proposed an anonymous password-authenticated key exchange protocol for the medical Internet of things (PAMI), which requires only a low-entropy password to achieve mutual authentication between a medical device and a telemedicine server to negotiate a high-entropy session key. Li et al. [62] proposed an unlinkable, collusion-resistant authenticated key agreement for VANETs. Cvitić et al. [63] examined Internet of Things (IoT) features for device classification, regardless of their functions. This type of categorization is required in a dynamic and heterogeneous environment, such as a smart home, where the quantity and types of devices are increasing regularly. A classification model is developed using the logistic regression approach. Mani et al. [64] presented the six most effective gradient-based adversarial attacks on the ResNet image recognition model. The authors suggested a unique ensemble defensive method based on the adversarial retraining technique.

### Research gap

In Table 1 we compare our contribution with the most relevant research papers which modeled software security attributes using the aspect-oriented technique. A review of the literature reveals that there is no evidence of authentication threats modeling in a mal sequence diagram that employs all the core features of the aspect-oriented technique. To do so, no security and AOM profile is created for the mal sequence diagram. The verification of the aspect-oriented mal sequence woven diagram is not evident from the literature. Besides that, the previous approaches have no support for the aspect OCL. Therefore, we aim to model and verify authentication threats mitigation in an aspect-oriented mal sequence woven model. Hence, two research questions (RQs) are addressed. The first question is to address the modeling problems, and the second question is to address the verification of the aspect-oriented mal sequence woven model. The research questions are given below.

**RQ 1.** How to model authentication threats mitigation in the aspect-oriented mal sequence model?
**RQ 2.** How to verify the correctness and completeness of the aspect-oriented mal sequence woven model?

**Table 1:** Summary table.

Paper #	Security attributes	Syntax and Semantics	Security profile	AOM profile	OCL constraints profile	Extensibility to Aspect OCL	Verification of aspect-oriented mal sequence woven diagram	AOM Concepts Used				
								Aspect	Advice	Join Point	Point cut	Weaving
**[25]**	Authorization	✓	-	-	-	-	-	✓	✓	✓	✓	✓
**[30]**	Authorization	-	-	-	-	-	-	✓	-	-	-	✓
**[31]**	Authentication and Authorization	-	-	-	-	-	Using Alloy Analyzer	✓	-	-	-	✓
**[33]**	Authentication	-	-	-	-	-	-	✓	✓	✓	-	✓
**Our Approach**	**Authentication**	✓	✓	✓	✓	✓	**Mathematically**	✓	✓	✓	✓	✓

## Proposed methodology

This section describes the AOM approach and methodology of our research.

### Authentication threats modeling

**Fig 2:**
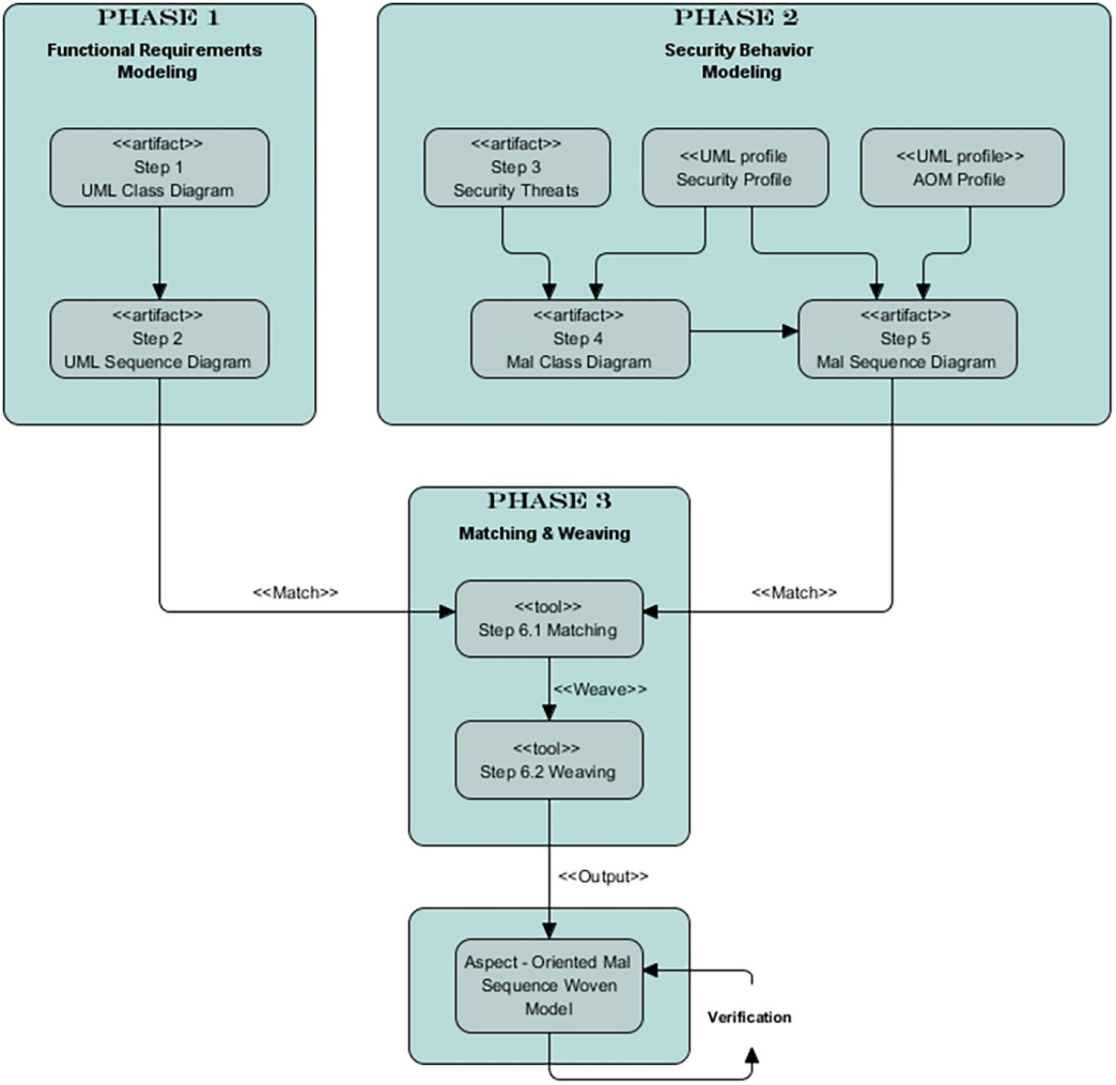
Research approach.

We present an AOM approach to cover the modeling of functional and security behavior as shown in Fig 2. Our key aim is to have an approach that supports security designers in modeling security solutions without changing the software’s core functionalities. This approach supports the UML class diagram, UML behavioral diagrams, and the security-specific mal diagrams with their respective UML profiles.

The approach can be divided into three main phases. The first phase comprises the modeling of functional requirements. The second phase comprises the modeling of the security behavior of software systems, and the third phase comprises the matching and weaving process. The eclipse tool is used for modeling all the phases of the approach. This tool requires a transformation language, i.e., Kermeta [65,66] or ATL [67] to transform the models into the woven model. The three phases are further divided into six steps to model the authentication. These steps are explained below.

Functional requirement modeling involves steps 1 and 2, security behavior modeling involves steps 3 to 5, and matching and weaving involve steps 6.1 and 6.2. Step 1 involves designing a class diagram of a system. This diagram is also referred to as the base class diagram. Step 2 involves the design of a sequence diagram. This sequence diagram is also referred to as the base sequence diagram. Step 3 identifies the potential threats to a software system. Step 4 entails modeling threats in a mal class diagram using the security profile. In step 5, the mal sequence diagram models the authentication mechanism as an aspect. Aspect is a separate modular unit that can be managed and modified independently. Step 6 involves the matching and weaving of the mal sequence diagram with the base sequence diagram. The result of matching and weaving is the aspect-oriented mal sequence woven model. The verification of this model is performed using mathematical theorems.

### Research methodology

#### Data collection

The data collected has two types of requirements, i.e. the functional requirements are taken from the ATM case study [68] and security requirements are collected from other sources [69–73]. The ATM case study is used for analysis and validation purposes. The ATM system permits people to do routine transactions such as withdrawing cash, transferring funds, etc. The ATM is always associated with an authorized bank and permits users to get their accounts and process transactions upon completion of the authentication process by the bank. For this purpose, the user uses the card and PIN. If the transaction is successful, the user gets the cash; otherwise, the ATM shows an error on the screen. All the steps are followed to model the ATM diagrams.

#### Experimental process

For the proposed research, we performed a control experiment, which is defined using the following attributes:

**Object of Study:** Aspect-oriented modeling is the main object of study in this experiment.

**Purpose:** Modeling and verification of authentication threats mitigation in aspect-oriented mal sequence woven model.

**Quality Focus:** Modeling and verification of AOM is the main focus of our experiment.

**Perspective:** The experiment is conducted from the perspective of a developer.

**Context:** The experiment is conducted in the context of software modeling.

The planning of this experiment can be defined using the following attributes. For both RQs, the null and alternate hypotheses with their variables are given.

**RQ.1.** How to model authentication threats mitigation in the aspect-oriented mal sequence model?
**Ho:** We can model authentication threats mitigation in the aspect-oriented mal sequence model.
**HA:** We cannot model authentication threats mitigation in an aspect-oriented mal sequence model.
  - **Dependent variable:** Woven model
  - **Independent variable:** Aspect, introduction, advice, join point, pointcut, matching, and weaving.
**RQ.2.** How to verify the correctness and completeness of the aspect-oriented mal sequence woven model?
**Ho:** We can verify the correctness and completeness of the aspect-oriented mal sequence woven model.
**HA:** We cannot verify the correctness and completeness of the aspect-oriented mal sequence woven model.
  - **Dependent variable:** Verification of correctness and completeness of the aspect-oriented mal sequence woven model.
  - **Independent variable:** Mathematical theorems

## UML profiling

### UML profiling

The first objective of our research is to create an aspect-oriented UML profile for the mal sequence diagram to model authentication threats and their corresponding mitigation. Therefore, in this section, we include the creation of a security profile, and an AOM profile using the UML extension guidelines.

#### Unified modeling language (UML)

The UML is widely used in modeling software systems [74]. Nowadays, the broadly used language for designing different object-oriented systems is UML [75,76]. It is considered a standard language [77] for many domains. It covers the core modeling of software and also provides an extension mechanism [78] to define new constructs for various domain problems. Various systems use the extension mechanism for defining and describing new modeling units, also known as modeling constructs. The extension mechanism delivers the common semantics to allow a UML metamodel to be extended and avoid any kind of semantic conflict.

The UML profile is mainly defined and constructed using three main concepts [79]. The first concept is a stereotype, which is represented by the class known as the profile class. The second concept is tagged values, which represent the stereotype’s properties, and the third concept is a constraint, which applies to a stereotype [80]. Likewise, the UML also provides design models that are easily extended for the aspect-oriented modeling (AOM) technique [81].

#### Profile attributes

The profile of extended elements consists of seven attributes. These attributes help to specify the extended element with features. In these attributes, the name, association, symbol, generalization, and constraints are essential for the element specification, while the operation and description, are not essential.

#### Eclipse tool

Eclipse is an open-source tool that provides an approachable environment, a GUI, friendly editors [82], and a drag-and-drop facility. This tool supports the design and the reusability [83] of all the UML diagrams.

### Security profile

Security profile consists of profile diagrams and their respective profile tables. These diagrams and tables specify the extended constructs for the mal class and mal sequence diagram.

#### Mal class diagram

**Fig 3:**
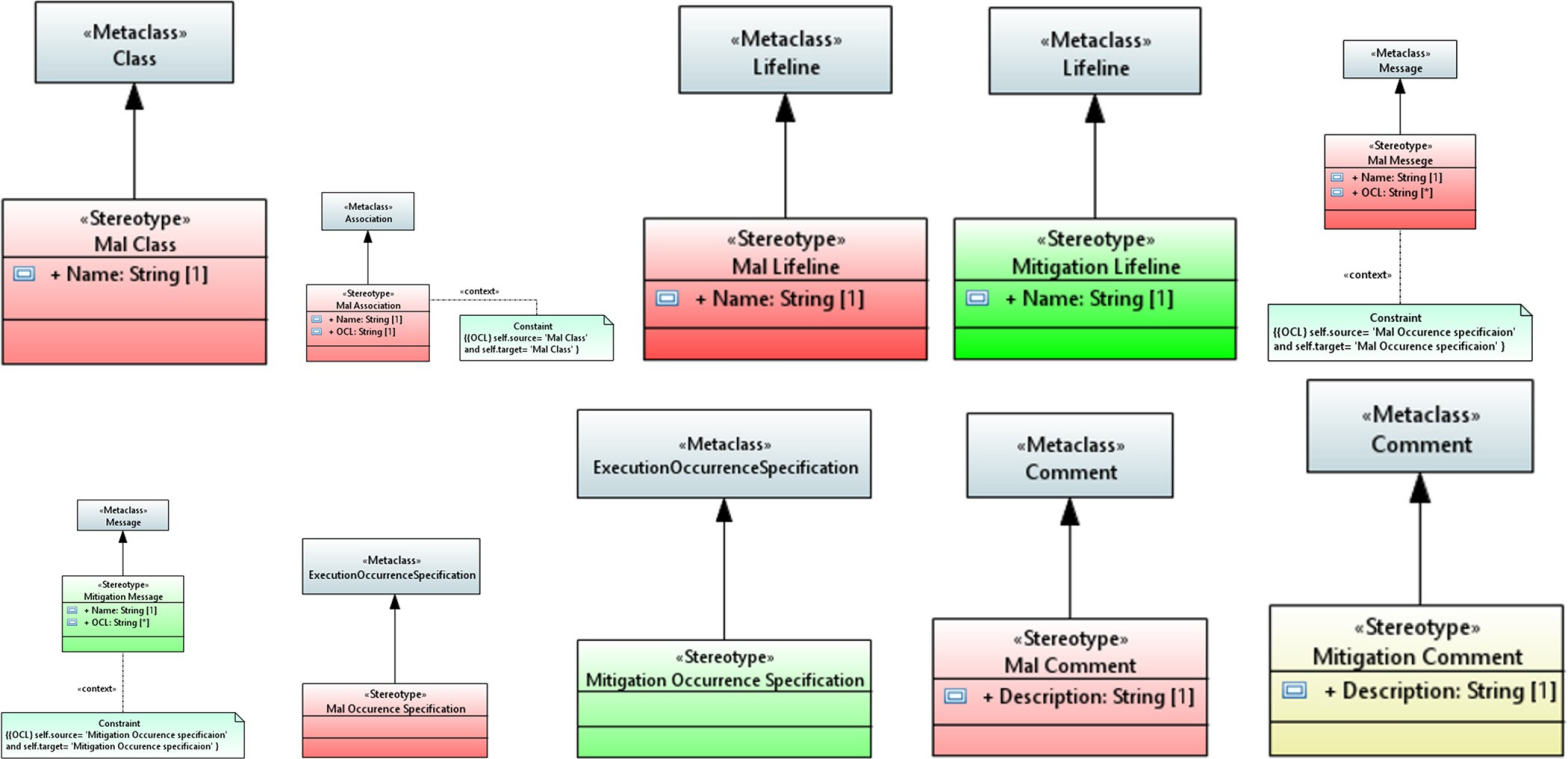
a. Mal class. b. Mal association. c. Mal lifeline. d. Mitigation lifeline. e. Mal message. f. Mitigation message. g. Mal occurrence specification. h. Mitigation occurrence specification. i. Mal comment. j. Mal comment.

The constructs, i.e., mal class and mal association, are extended from the UML metaclasses to model security in the mal class diagram. Mal class and mal association are stereotypes, as shown in Fig 3A and 3B. The mal class and mal association must have a specific name, and the source and destination of mal association must be mal class as defined by the constraint. The profile tables for the mal class and mal association are given in Table 2A and 2B, respectively. These profiles are created using the UML extension mechanism

**Table 2:** a. Mal class. b. Mal association. c. Mal lifeline. d. Mitigation lifeline. e. Mal message. f. Mitigation message. g. Mal occurrence specification. h. Mitigation occurrence specification. i. Mal comment. j. Mitigation comment.

**Name: Mal class**
**Description:** The mal class is a construct to specify malicious entities. The red color shows that this class is harmful to the normal classes of the system.
**Diagram**: The UML metaclass extends the mal class to use in the mal sequence diagram. • Semantics: Mal class refers to a class that defines mal entities. • **Notation**: The mal class represents by a square-like symbol. Mal class consists of three portions. The upper portion of the class is used for the class name, the second portion is used for the attributes, and the third portion is used for methods.
**Generalization:** Class
**Association:** Mal [1..*] Mal class
**Operation**: No operation
**Constraint**: No constraint is applied to the mal class.
**Name: Mal association**
**Description:** The mal association is a construct to use as a relationship between mal classes. The red color shows a threat relationship between the mal classes.
**Diagram**: The UML metaclass extends the mal association to use in the mal class diagram. • Semantics: It refers to a relationship between mal classes. • **Notation**: Mal association represents by a line-like shape.
**Generalization**: Association
**Association**: Mal [1] Mal class
**Operation**: No operation
**Constraint**: The constraint that applies to the mal association is given below. The source and target of mal association is mal class. self.source= ’Mal Class’ and self.target= ’Mal Class’
**Name: Mal lifeline**
**Description:** Mal lifeline specifies the malicious objects or entities in a mal sequence diagram.
**Diagram**: The UML metaclass extends the mal lifeline to use in the mal sequence diagram. • Semantic: It refers to an object or entity in a mal sequence diagram. • **Notation**: The lifeline represents by a box-like shape, and a dotted line (always vertical) connected to the center of the box’s bottom.
Generalization: Lifeline
**Association**: MessageName[1..*] Mal Message
**Operation**: No operation
**Constraint**: No constraints
**Name: Mitigation lifeline**
**Description:** Mitigation lifeline specifies objects or entities in a mal sequence diagram.
**Diagram**: The UML metaclass extends the mal lifeline to use in the mal sequence diagram. • Semantic: It refers to an object or entity in a mal sequence diagram. • **Notation**: The mitigation lifeline represents by a box-like shape, and a dotted line (always vertical) connected to the center of the box’s bottom.
Generalization: Lifeline
**Association**: MessageName[1..*] Mitigation Message
**Operation**: No operation
**Constraint**: No constraint
**Name: Mal message**
**Description:** The mal message specifies the threat message between mal lifelines in the mal sequence diagram.
**Diagram**: The UML metaclass extends the mal message to use in the mal sequence diagram. • Semantic: It refers to a specific message that carries a threat between mal lifelines. • **Notation**: An arrow-like shape uses for the mal message.
Generalization: Message metaclass
**Association**: MalMessage[1] Mal lifeline
**Operation**: No operation
**Constraint**: The constraint that applies to the mal message is given below. The mal occurrence specification specifies the source and target of the mal message. self.source= ’Mal Occurrence Specification’ and self.target= ’Mal Occurrence Specification’
**Name: Mitigation message**
**Description:** The mitigation message specifies the prevention message between lifelines in the mal sequence diagram.
**Diagram**: The UML metaclass extends the mitigation message to use in the mal sequence diagram. • Semantic: It refers to a message between lifelines to prevent the threat. • **Notation**: An arrow-like shape uses for mal message.
Generalization: Message metaclass
**Association**: Mitigation message[1] Mitigation lifeline
**Operation**: No operation
**Constraint**: The constraint that applies to mal message is given below. The mitigation occurrence specification specifies the source and target of the mitigation message. self.source= ’Mitigation Occurrence Specification’ and self.target= ’’Mitigation Occurrence Specification’
**Name: Mal occurrence specification**
**Description:** It shows the activation of a specific mal object in a mal sequence diagram.
**Diagram**: The metamodel extends the mal occurrence specification to use in the mal sequence diagram. • Semantics: It refers to an activation of a mal object uses in a mal sequence diagram. • **Notation**: Mal occurrence specification represents a shape that resembles a vertical rectangle and is placed over the lifeline or on another mal occurrence specification.
**Generalization**: Execution occurrence specification meta-class.
**Association**: MalMessage [1..*] MalMessage
**Operation**: No operation
**Constraint**: No constraint
**Name: Mitigation occurrence specification**
**Description:** It shows the time interval or activation of a specific object used in mitigation in a mal sequence diagram.
**Diagram**: The metaclass extends the mal occurrence specification to use in the mal sequence diagram.. • Semantics: It refers to a time interval for an object in the mal sequence diagram as long as the bar is active. • **Notation**: It represents by a shape that resembles a vertical rectangle and is placed over the lifeline or on another occurrence specification. The green color shows the activation of an object to prevent the attack.
**Generalization**: It is generalized from the occurrence specification metaclass.
**Association**: MitigationMessage [1..*] MitigationMessage
**Constraint**: No constraints
**Name: Mal comment**
**Description:** It shows the textual description of an element in a model.
**Diagram**: The metaclass extends the mal comment to use in the mal class or mal sequence diagram. • Semantics: It refers to a textual annotation used for an element in a mal class diagram. • **Notation**: It represents by a rectangle with a bent corner.
**Generalization**: Comment metaclass.
**Association**: The association of mal comment with mal class, mal association, and mal message is given below MalComment [0 …*] Mal class, MalComment [0 …*] Mal association, MalComment [0 …*] Mal message
**Operation**: No operation
**Constraint**: No constraint
**Name: Mitigation comment**
**Description:** It shows the textual description of an element in a model.
**Diagram**: The metaclass extends the mal comment to use in the mal sequence diagram. • Semantics: It refers to a textual annotation used for an element in a mal class diagram. • **Notation**: It represents by a rectangle with a bent corner.
**Generalization**: Comment metaclass. **Generalization**: Comment metaclass.
**Association**: MitigationComment [0 …*] Mitigation message
**Operation**: No operation
**Constraint**: No constraint

#### Mal sequence diagram

Mal sequence diagram models the mitigation mechanism using the extended elements. The profile diagrams are given in Fig 3C–3J for the extended elements. The profile tables are given in Table 2C–2J, which are created using the UML extension mechanism.

### Object constraint language (OCL)

**Fig 4:**
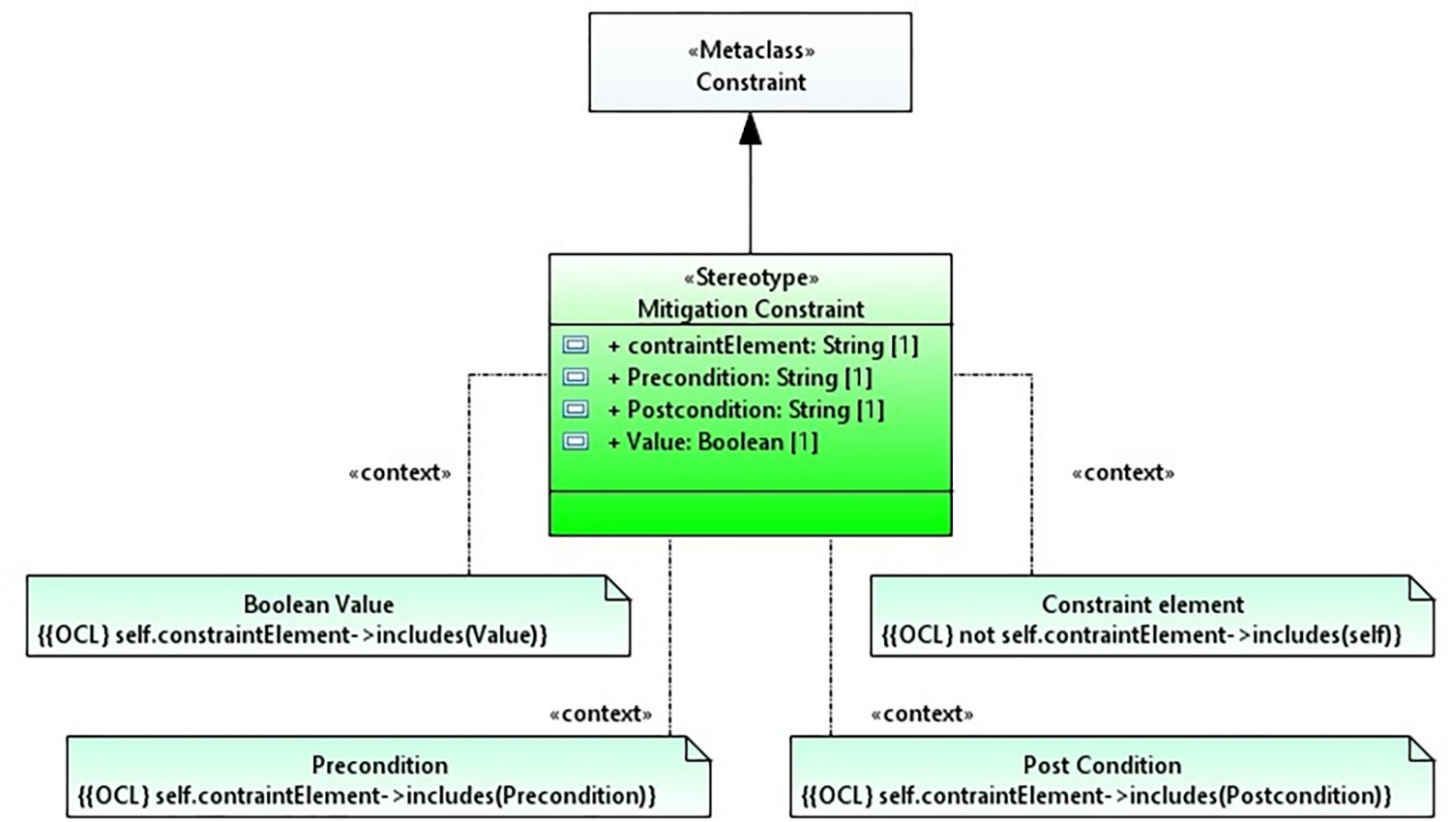
Mitigation constraint.

We use an object constraint language to specify constraints on the UML structural and behavioral models. OCL is a standard language used for constraint specification [84] in the area of modeling and is considered a significant part of model-driven engineering. In practice, it is used in industries and academia to define constraints in the form of expressions [85]. OCL has the advantage that it places constraints on the models [86] that can easily be understood by modelers. Another significant benefit of this language is that it is independent of programming languages [87] and has no side effects when used in UML models [88,89]. The aspect OCL [90,91] is the extension of OCL that provides a solution for specifying crosscutting constraints separately as an aspect. However, in this research, we only propose the extension of mitigation constraints. The mitigation constraint is extended from the UML metaclass to specify security constraints as shown in Fig 4. Table 3 shows the profile table.

**Table 3:** Mitigation constraint.

Name: Mitigation constraint
**Description:** Mitigation constraint specifies the constraints on the stereotypes and their operations.
**Diagram**: The constraint metaclass extends the mitigation constraint to define the constraints in the mal sequence diagram. • **Semantics:** It refers to the expression that performs operations on the elements of the model. It shows a restriction that must be valid. • **Notation:** It represents by a rectangle with a bent-corner shape labeled with {{OCL true}.
**Generalization:** Constraint
**Association:** Mitigation constraint [0..*] Mitigation message
**Constraint:** The four constraints that apply to the mitigation constraint are given below. a. A constraint cannot be applied to itself. not self.contraintElement→includes(self) b. Constraint must have some precondition. self.contraintElement→includes(precondition) c. Constraint must have some post-condition. self.contraintElement→includes(postcondition) d. The value specification for a constraint must evaluate to a boolean value. self.constraintElement→includes(value)

### Aspect-oriented modeling (AOM)

In this section, we created the profile for AOM features used in the mal sequence diagram. These AOM features play a key role in localizing the concern.

#### AOM features

**Aspect—**Aspect models the crosscutting behavior of concern. Aspect is separately managed from the system base models and is considered a unit that encloses the properties of concern [92].

**Advice—**Advice shows the behavior of concern. It modifies the behavior of base models when applies to the specified base models’ join points. The advice adds behavior to the base dynamic models, e.g. sequence diagrams. It has three types.

1. **Before Advice:** Addition of advice before the join point of the base model.
2. **After Advice:** Addition of advice after the join point of the base model.
3. **Around advice:** Substitution of the base model’s join point by advice.

**Join point—**In the context of AOM, all UML core elements that are used for modeling are considered join points. For example, the message is a core element that is considered a join point in a sequence diagram.

**Pointcut—**A pointcut is an expression that uses textual language or OCL to apply the advice on the selected join points. A pointcut is used for the matching process.

**Matching—**The matching process matches the join points of base models with the join points specified by a pointcut in aspect [19].

**Weaving -** The weaving process weaves or injects the aspect into the base models.

**Adaptation—**The aspect uses adaptation. Adaption [19] has two types of rules, i.e., addition and removal. The addition rule adds the aspect to the base model, while the removal rule, removes the element from the base model.

#### AOM profile

**Fig 5:**
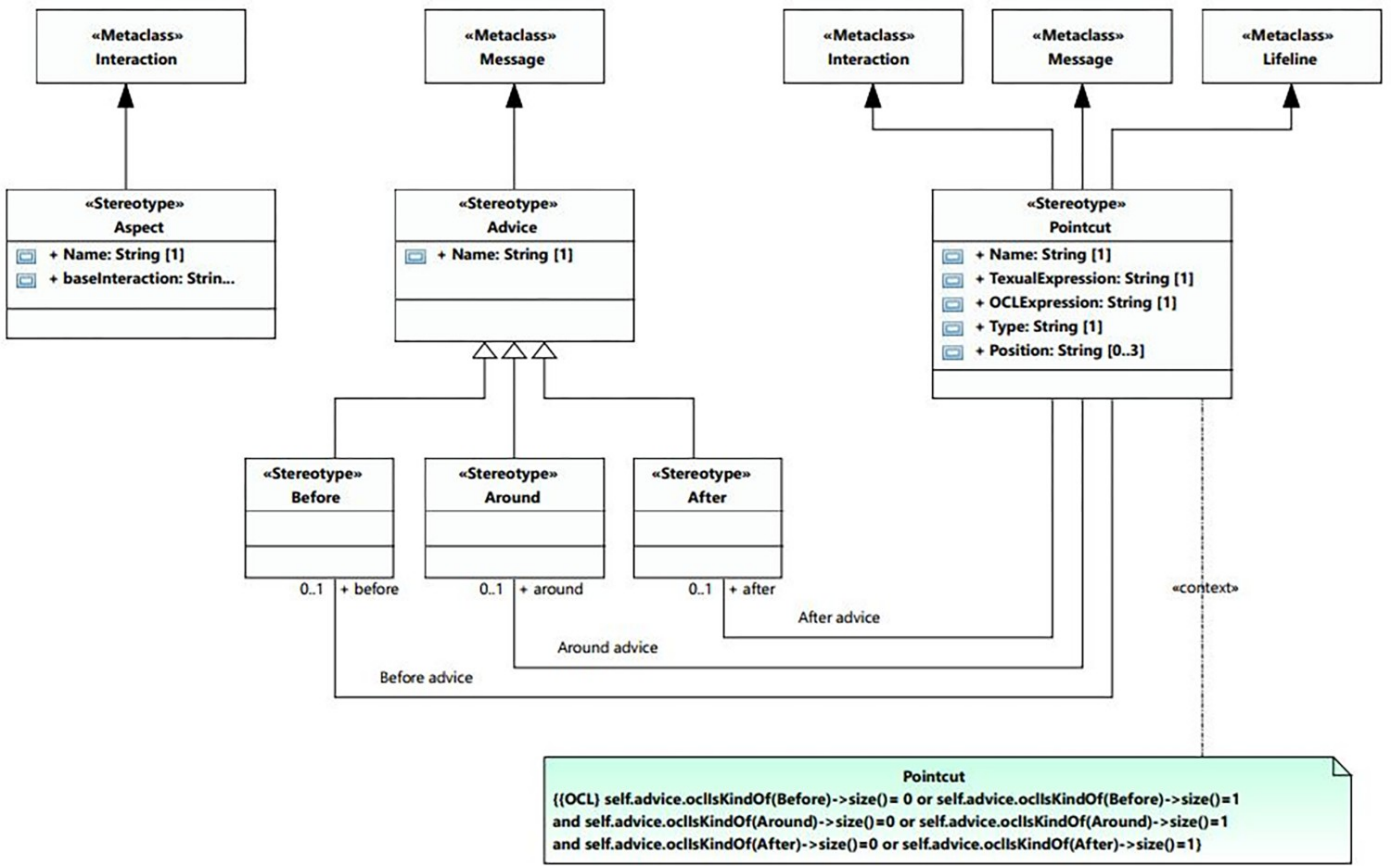
AOM profile diagram.

The profile diagram in Fig 5 shows the constructs of the aspect-oriented technique. These constructs are extended from the UML metaclass. The aspect and advice are derived from interaction and message, respectively, and the pointcut is derived from three metaclasses. The pointcut is associated with the advice. The AOM profile is given in Table 4 which is created using the UML extension mechanism.

**Table 4:** AOM profile table.

Name	Generalization	Extension	Association
Aspect	None	UML:: Interaction	None
Advice	None	UML:: Message	pointcut[1]:Pointcut
Before	Advice	The message metaclass extends the before stereotype.	pointcut[1]:Pointcut
After	Advice	The message metaclass extends the after stereotype.	pointcut[1]:Pointcut
Around	Advice	The message metaclass extends the around stereotype.	pointcut[1]:Pointcut
Pointcut	None	UML:: Lifeline UML:: Message UML:: Interaction	Before advice[0..1]:Before, After advice[0..1]:After, Around advice[0..1]:Around,

### Analysis and results

Our second objective is to model and mitigate authentication threats using the aspect-oriented modeling technique. This objective is achieved through solving RQ. 1 given below. Using the ATM case study, we established the applicability and soundness of the proposed approach for security modeling. All the steps of the approach are followed in the case study.

RQ 1. How to model authentication threats mitigation in the aspect-oriented mal-sequence model?

#### ATM case study

The data collected has two types of requirements, i.e., the functional requirements are taken from the ATM case study and security requirements are collected from other sources. The ATM case study is used for analysis and validation purposes.

##### Model a class diagram

**Fig 6:**
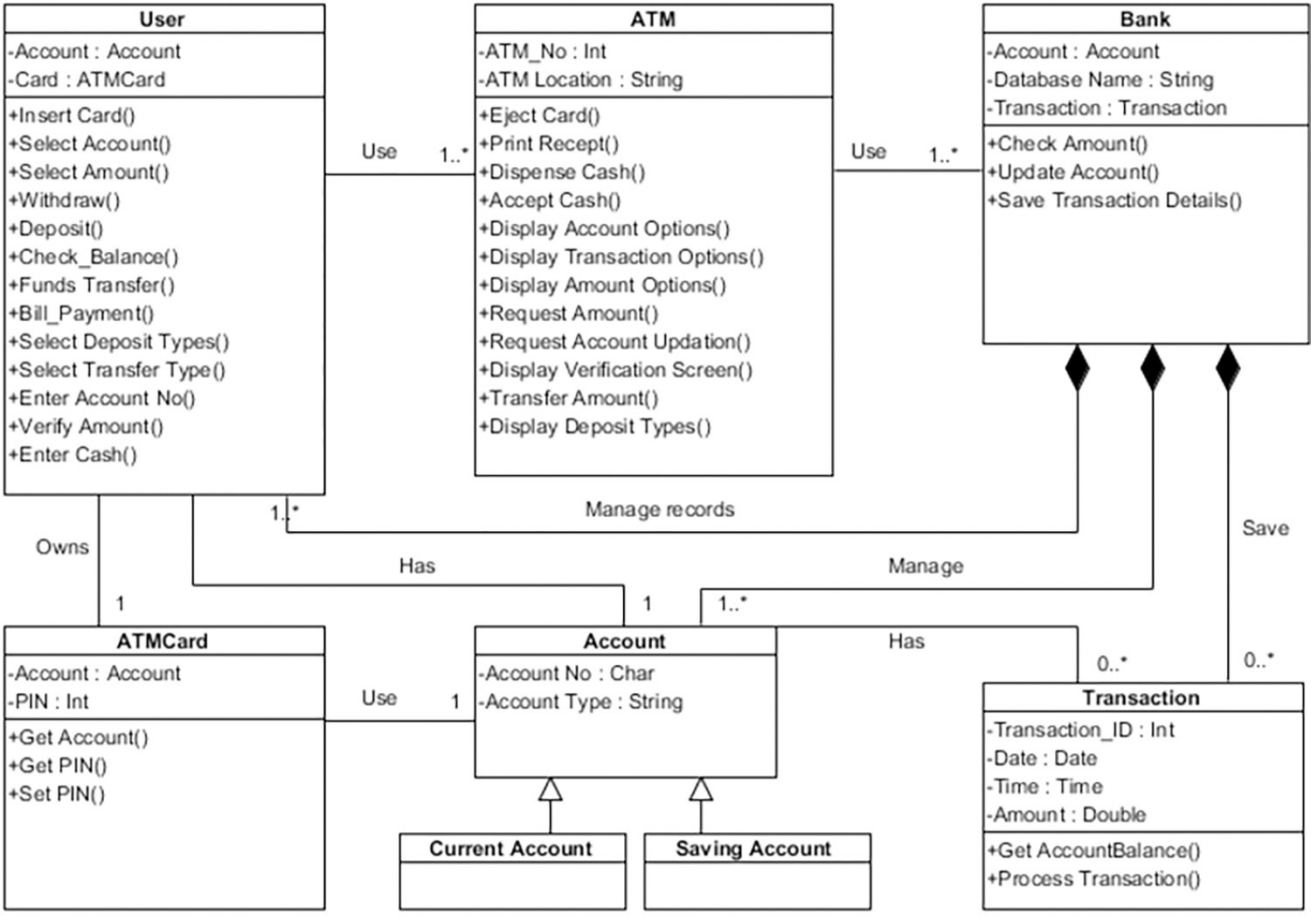
Class diagram.

In this step, the structural view of an ATM system using a class diagram is modeled as shown in Fig 6. The structural view shows various classes and the associations between them. In the class diagram, the user class performs several operations, i.e., withdrawing cash, balance inquiry, depositing cash, etc. The user uses the ATM card for transactions. The ATM class performs its operations, i.e., displaying various options to the user, accepting and withdrawing cash, etc. The ATM is associated with the bank to perform transactions for the user.

##### Model a sequence diagram

**Fig 7:**
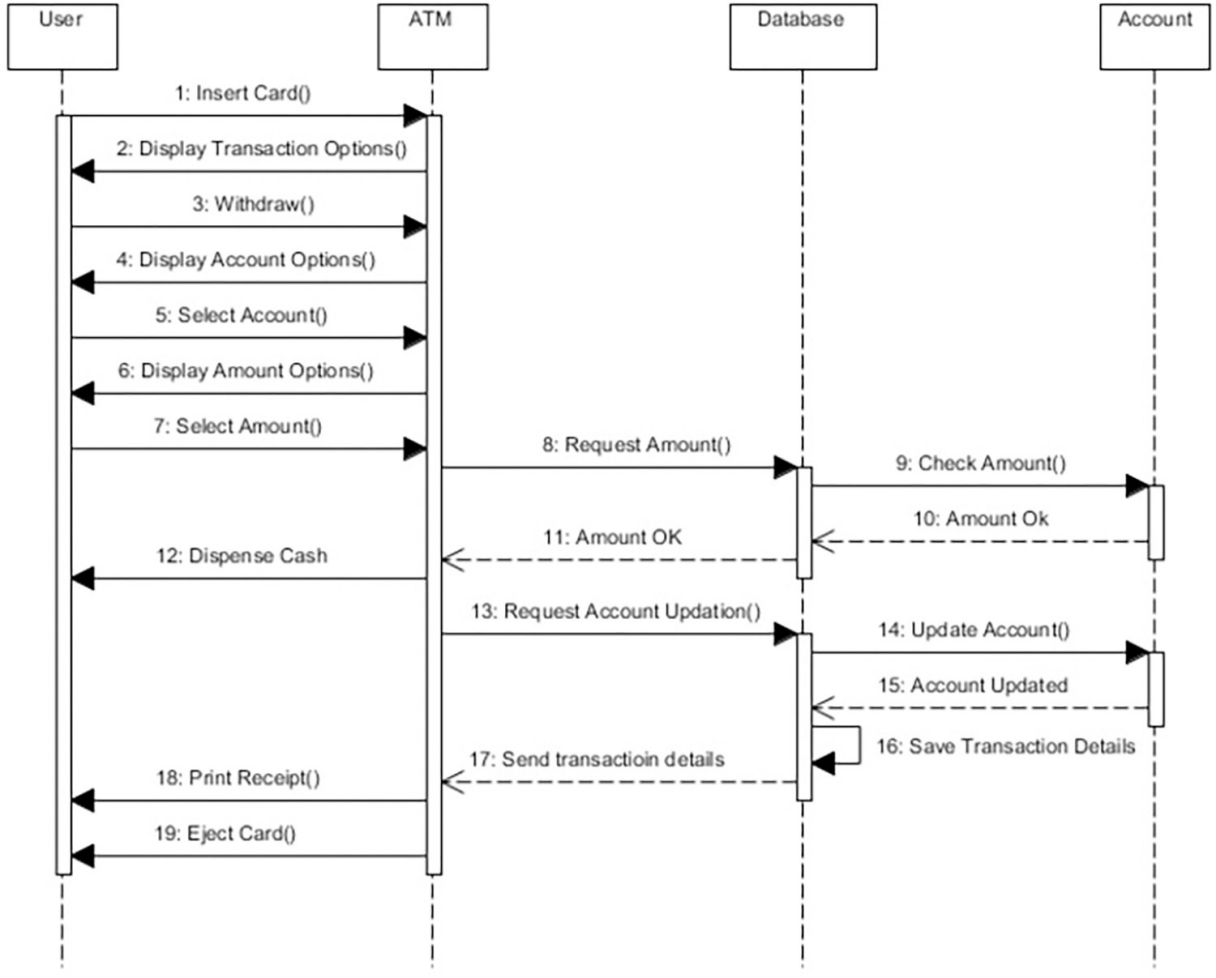
Sequence diagram.

In this step, the sequence diagram is modeled to show the sequence-wise behavior of objects. This diagram is referred to as a base sequence diagram. The sequence diagram for the withdrawing cash is modeled as shown in Fig 7. Similarly, we can model other scenarios such as depositing cash, transferring funds, etc., using the sequence diagram.

##### Security threats identification

In this step, the most important task is to identify the potential security threats to the ATM system. These threats exist in the form of various frauds and attacks. For fraud to happen, hackers use different methods to achieve their malign purpose. The most common threats to ATM systems are card skimming and card trapping. The description of each ATM threat is given below.

**ATM Card Skimming**–Card skimming is the most popular threat to ATM systems. The hackers always use a device named a card skimmer, which resembles an ATM scanner, to take someone’s credit card data. When this device is completely planted over a card slot area of the ATM, it successfully scans and stores the card’s vital data as soon as the card is swiped by the user. This data is further used by the hacker to access the account and make transactions. The camera is also used and is often installed secretly in such a way that it can easily record the PIN entered by the user. The PIN is used along with the card information by the fraudsters.

**ATM Card Trapping**—In this fraud, the hackers install a device directly into or over the card area of the ATM. In this situation, the card is physically captured by the trapping device as soon as it is inserted. The captured card is retrieved by the fraudster to make transactions.

##### Model a mal class diagram

**Fig 8:**
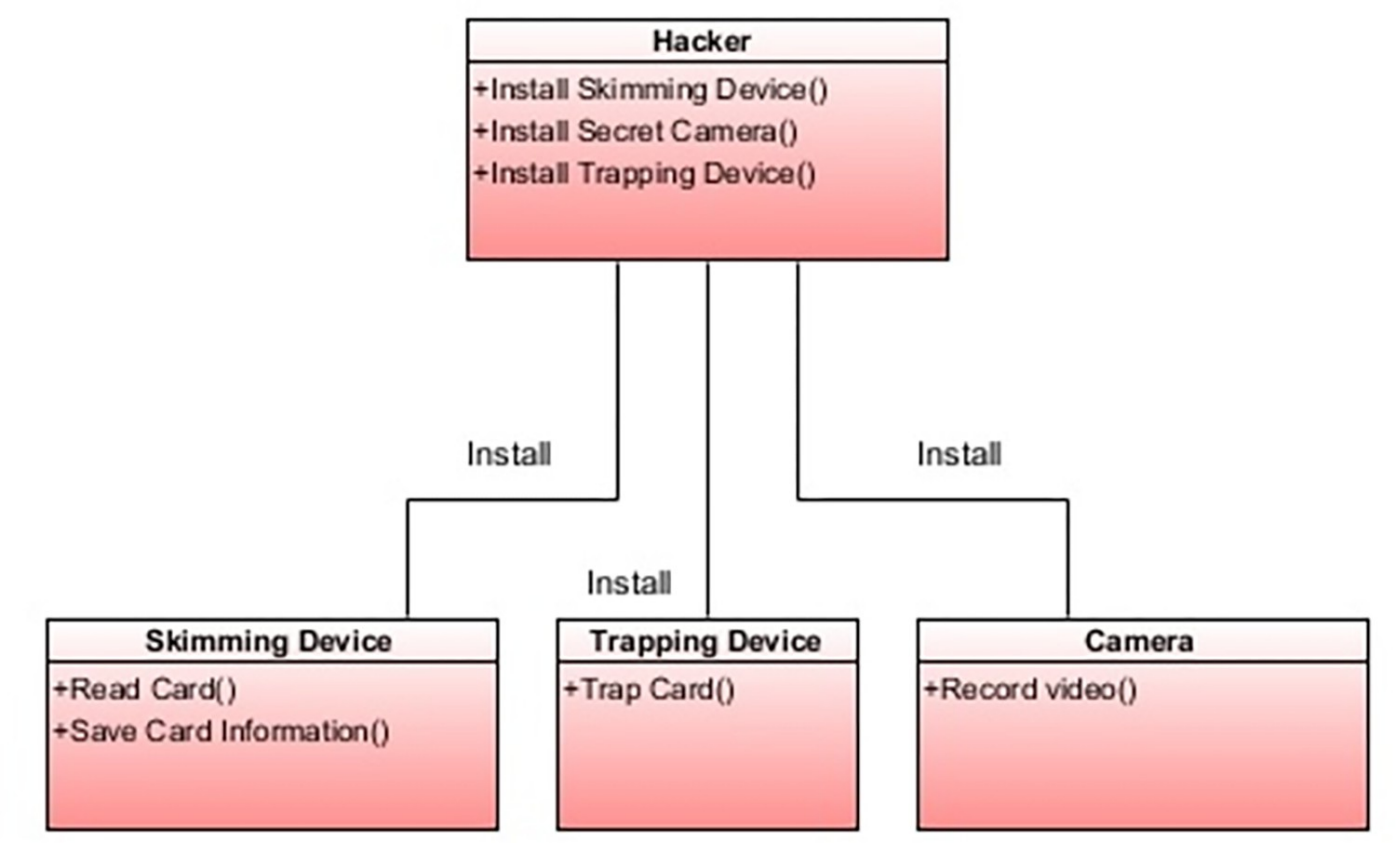
Mal class diagram.

In this step, the mal class diagram models the security threats as shown in Fig 8. The red color shows that these are malicious classes. A relevant study [1] used the MARTE profile to model the faults of a video conferencing system (VCS) in a class diagram. The author [31] also used the class diagram to model the threats. Therefore, the class diagram is used to show the static view of threats. In our case, the mal class uses the security profile to model the security threats. The mal class defines different operations that are responsible for the attack on the ATM system and how the mal classes are linked with each other to perform an attack.

##### Model a mal sequence diagram

In this step, the practical mitigation of the two security threats is modeled in the mal sequence diagram using the aspect-oriented technique. Multi-factor authentication (MFA) is used as a mitigation mechanism to prevent security threats. It is a kind of authentication [73] to guarantee and ensure that the user who wants to access the system is a real one, i.e., it ensures that only the authentic user should be allowed and the one who is not the user of legal property must be barred. The validity of the user is checked with different mechanisms and practices [93] to facilitate the protection of vital user data [94]. For better authentication, we consider a three-step approach which is a form of multi-factor authentication. The steps of this approach are given below.

**Step 1:** The user is authenticated physically based on an ATM card.
**Step 2:** The user is authenticated based on their PIN.
**Step 3:** The user is authenticated based on fingerprints.

**Fig 9:**
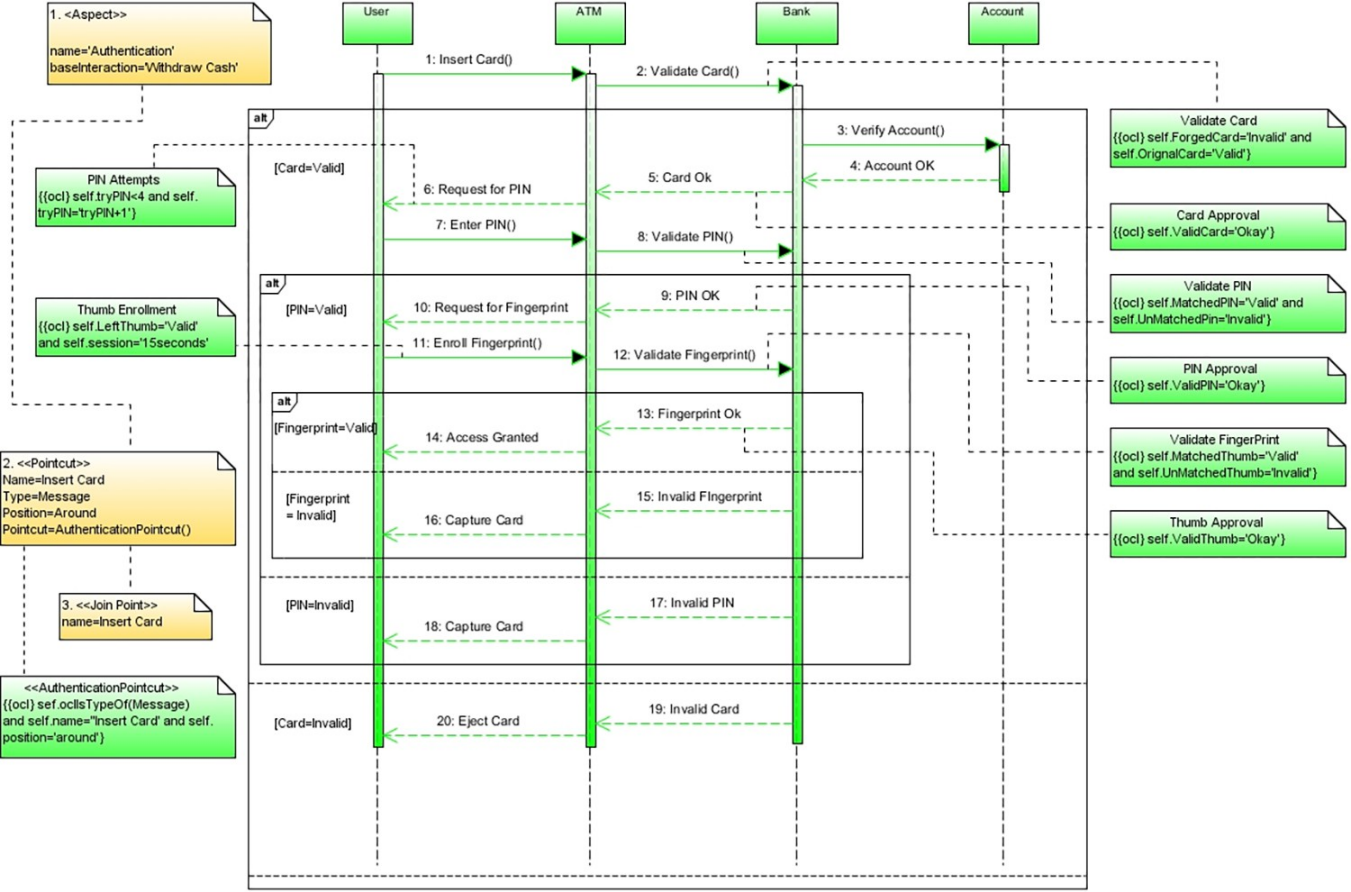
Mal sequence diagram.

Fig 9 the mitigation mechanism is modeled in mal sequence diagram. This diagram is also referred to as an authentication aspect. In the diagram, the join point is insert card, while the pointcut is expressed both in textual and in OCL form to add the aspect to the base sequence diagram. OCL constraints on different messages of the model are applied, which must be true to prevent any kind of threat. The diagram uses the security profile and the AOM profile to modularize the security.

##### Weaving models

In this step, the matching and weaving of the authentication aspect with the base sequence diagram are performed using semantics and algorithms. The output of this step is a woven model.

**Fig 10:**
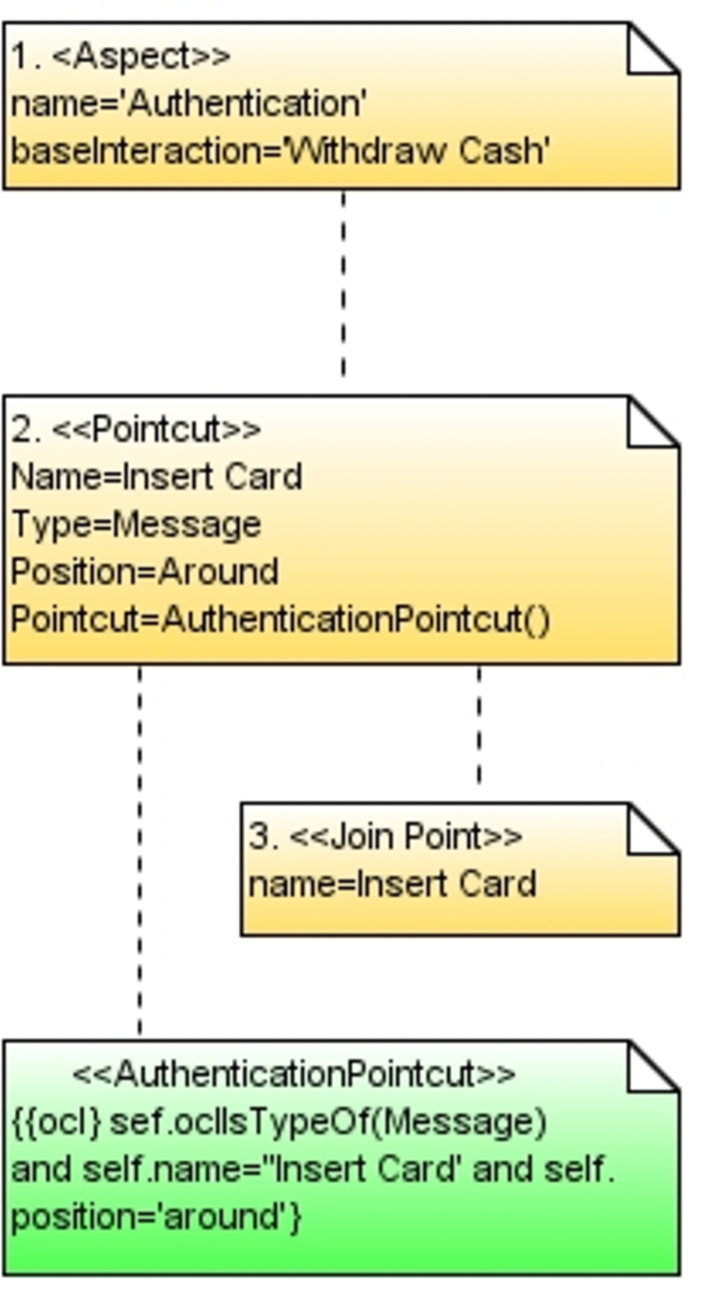
Matching process.

**Matching—**The matching process uses the matching algorithm to take the base sequence diagram’s join point and the join point specified in the pointcut as input. Fig 10 shows the matching process, where the join point is the insert card. In both models, the name of the join point is the same. Therefore, the matching process is successful.

**Fig 11:**
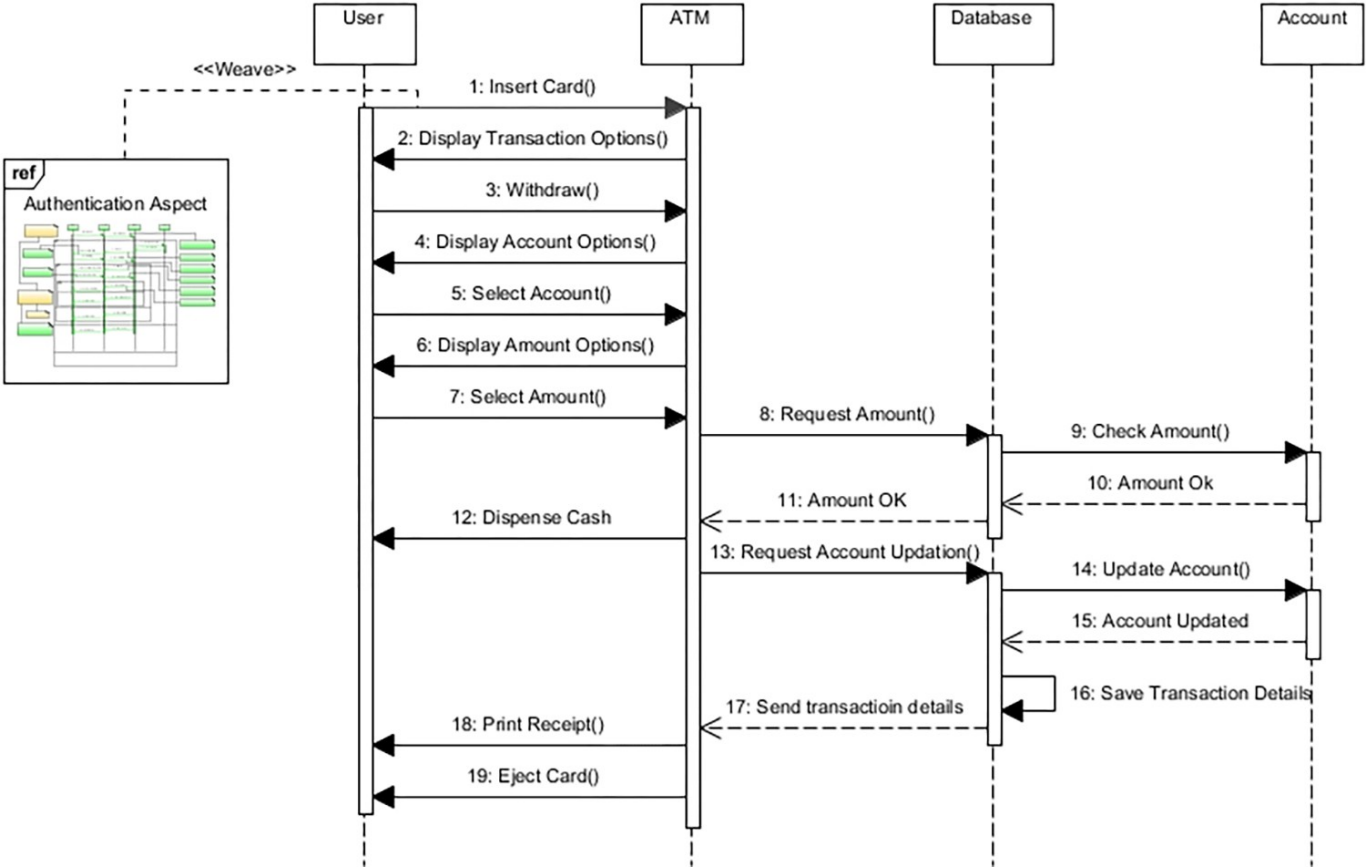
Weaving process.

**Fig 12:**
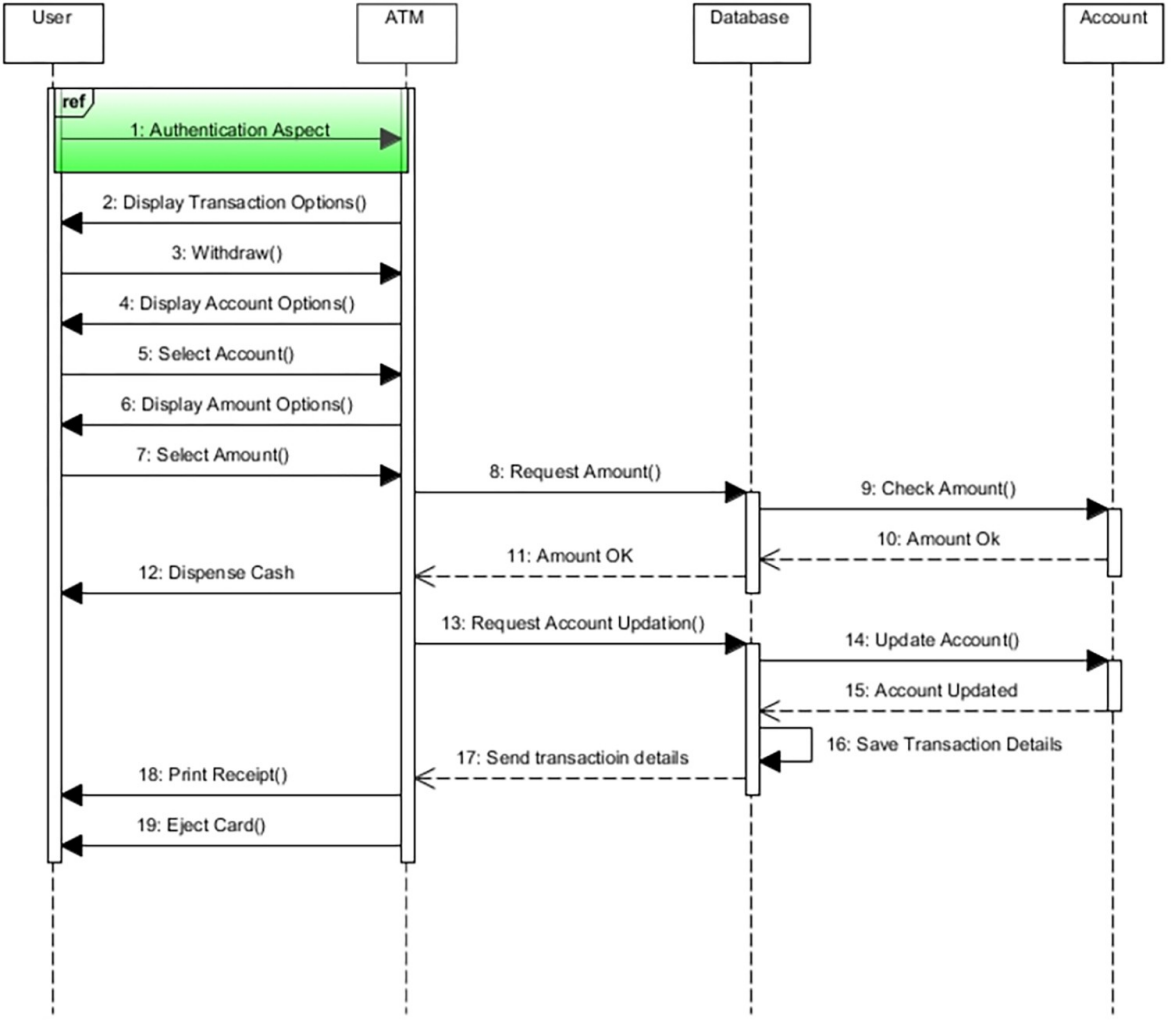
Aspect-oriented mal sequence woven model (woven model).

**Weaving—**In this step, the weaving process is performed as shown in Fig 11. The aspect model in Fig 9 is weaved into the base sequence diagram in Fig 7 to form a woven model in Fig 12. The ref is the reference to the actual model of authentication aspect. When the user wants to do a transaction, the reference will take the user directly to the authentication module, and after successful authentication, the ATM will start a step-by-step transaction for the user.

### Results

The following results are concluded from the ATM case study.

**Fig 13:**
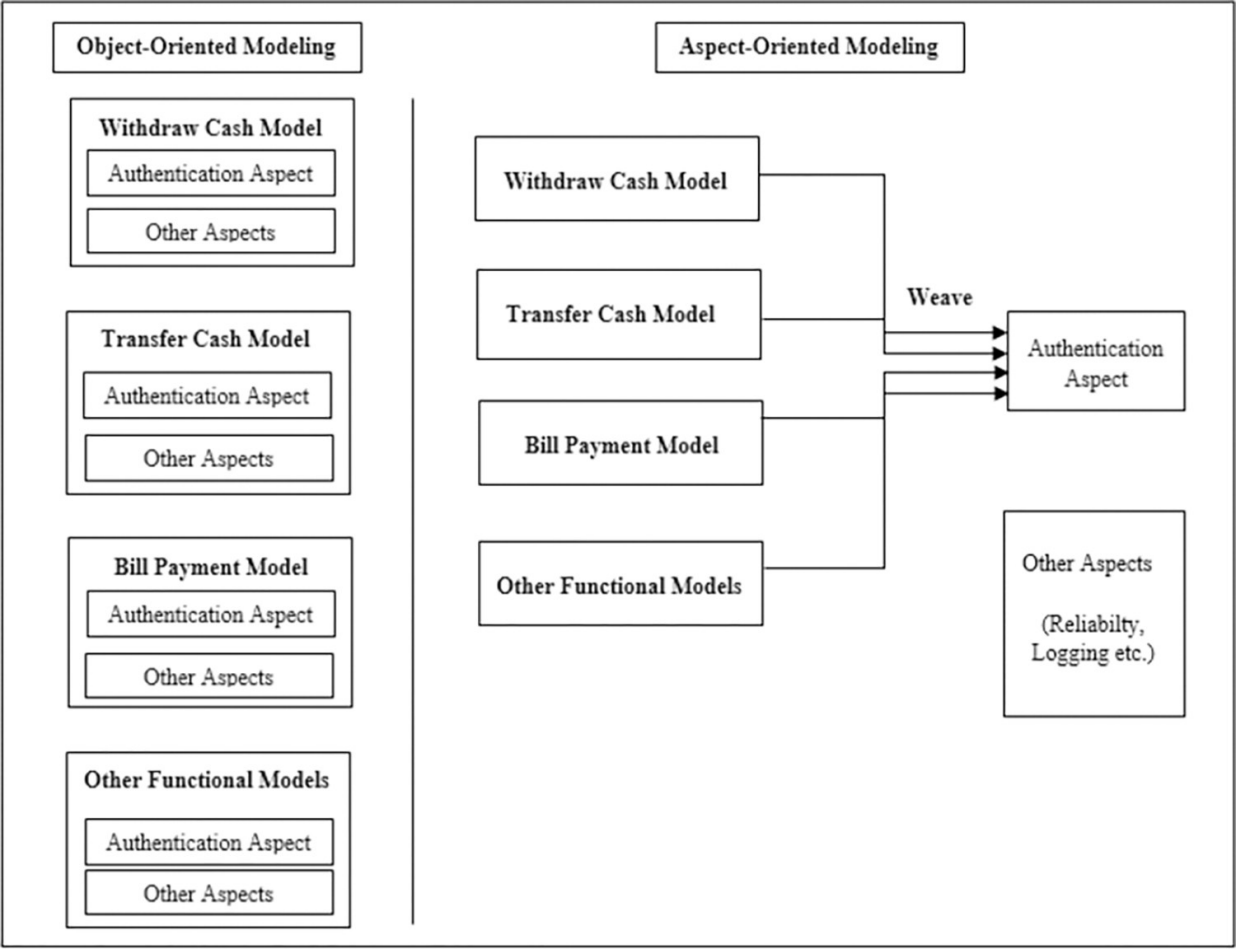
Comparison.

1. Using the security and AOM profiles, we successfully modeled the security threats and authentication mechanism in the mal sequence diagram.
2. The aspect and the withdraw cash sequence diagram are modeled separately from each other. Using the matching and weaving processes, we successfully injected the authentication aspect into the withdraw cash model to create the woven model. Similarly, we can model the ATM bill payment, and transfer cash model, and can weave the authentication aspect using the matching and weaving process.
3. If we compare both techniques as shown in Fig 13, we conclude that using the object-oriented technique, we have to model the authentication in every base sequence diagram, while using the aspect-oriented technique, we model the base sequence diagram and aspect separately and weave the aspect with the base model. In this way, the scattering and tangling are removed from the base sequence diagram using the aspect-oriented technique.

Thus, the null hypothesis for RQ.1 is correct because we successfully modeled authentication threats mitigation in the aspect-oriented mal sequence model.

#### Model verification

Our third objective is to verify the resultant woven model in terms of correctness and completeness. The RQ2 given below is about the verification of the woven model produced by the matching and weaving process. The verification of the model is performed in terms of correctness and completeness using mathematical theorems.

**RQ2.** How to verify the correctness and completeness of the aspect-oriented mal sequence woven model?

### Syntax

**Fig 14:**
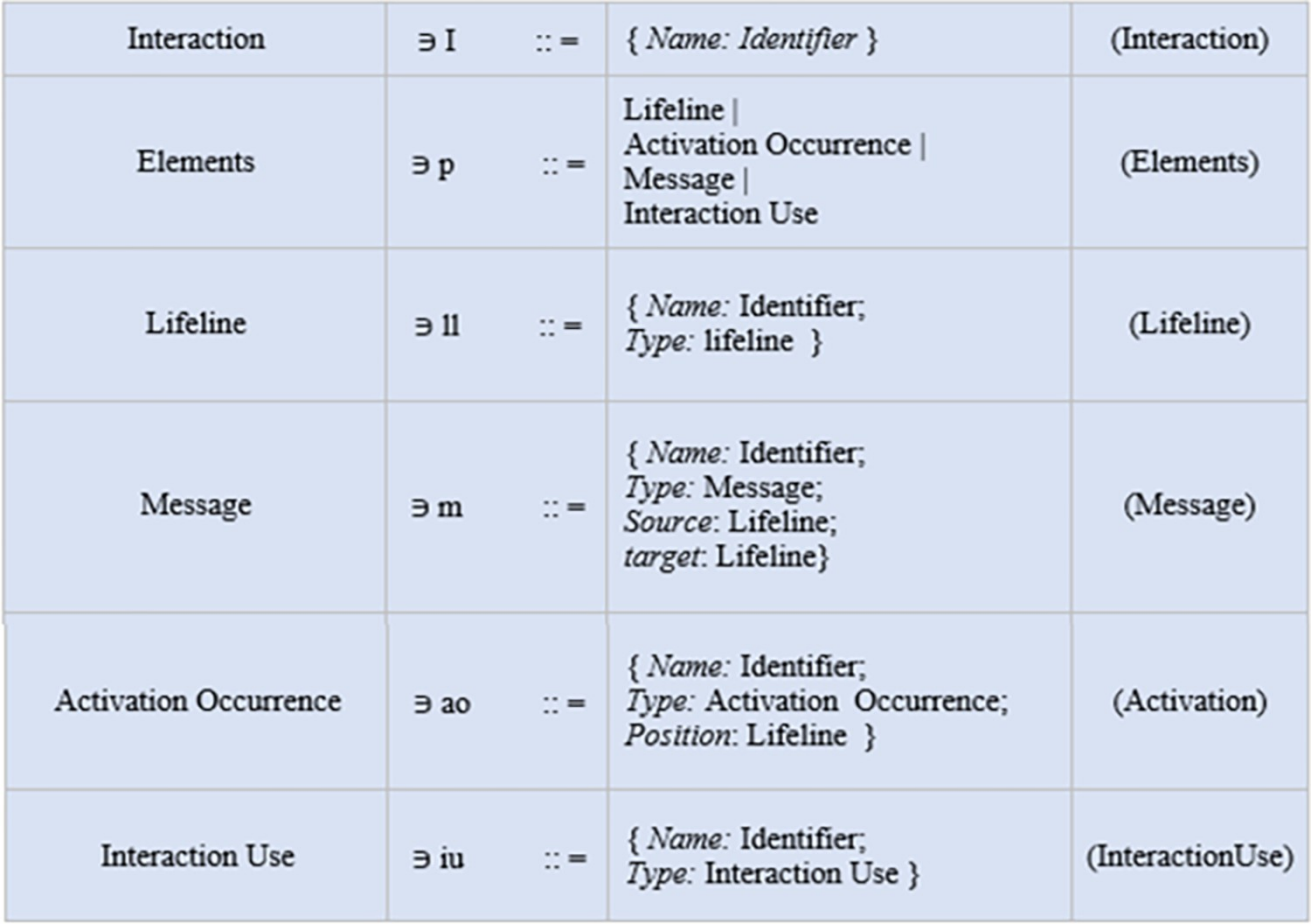
Sequence diagram syntax.

**Fig 15:**
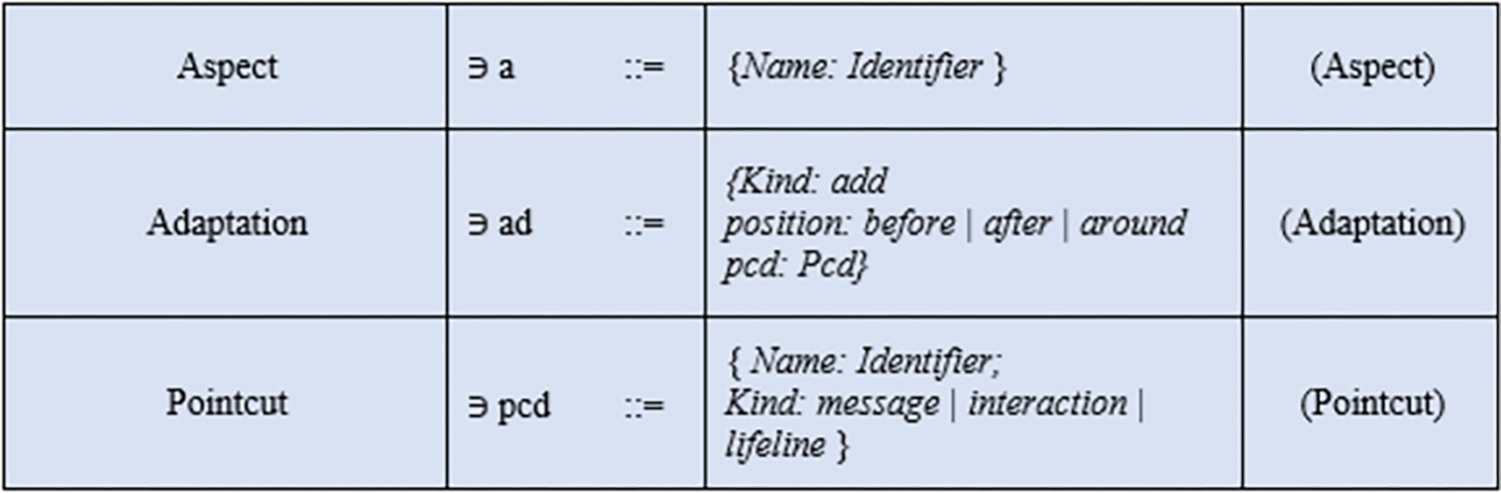
Aspect syntax.

In Fig 14, the syntax [25] used in the base sequence diagram and the mal sequence diagram is shown. The syntax covered only those core constructs that are mostly used in sequence diagrams. In Fig 15, the syntax of AOM features is shown. The adaptation used in the case study is of the kind "add" because the aspect is added to the base sequence diagram using a pointcut expression. Pointcut selected a join point is of kind "message" with an adaptation position of "around".

### Semantics

For model verification, two types of semantics, i.e., matching and weaving semantics, are used. The matching semantics defines a procedure to determine the join point selected by the aspect diagram using a pointcut expression, while the weaving semantics defines how to apply the aspect diagram at the selected join point of the base sequence diagram.

#### Semantic matching

**Fig 16:**
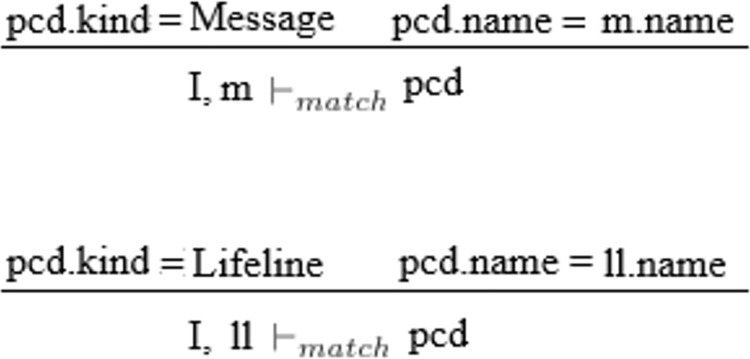
Matching semantics.

The semantic rules used for the matching process of the base sequence diagram and the aspect diagram are shown in Fig 16. In the figure, the m and II represent lifeline and message respectively which are the join points in the interaction I. The interaction is used for the base sequence diagram while the join points are the elements of the base sequence diagram. Pointcut pcd is used as an expression for matching between the base sequence diagram and aspect.

The expressions I, m ⊢ match pcd and I, II ⊢ match pcd show that matching matches the join point of interaction with the join point defined in the aspect’s pointcut. The expression pcd.kind = message shows that the join point used in the pointcut is of the kind message while the expression pcd.name = m.name shows that the name of the join point defined in the pointcut must be matched with the name of the message. The same is true for another expression.

#### Weaving semantics

The weaving semantics is defined by the pattern expressed in Eq (a). The state determines the position of weaving. Eq (b) shows the state before weaving, while when the weaving of models completes, the state changes to the end as shown in Eq (c), therefore this expression shows the state after weaving.

Pattern = (Interaction I, aspect a, join point p, state)        (a)

Before weaving = (I, a, p, weaving)                        (b)

After weaving = (I’, a’, p’, end).                                (c)

Hence, the expression (d) shows the transformation of the weaving process of the models.

(I, a, p, weaving) → (I’, a’, p’, end)                        (d)

### Algorithms

The algorithms used for matching and weaving processes in the ATM case study are given below.

#### Matching algorithm

The algorithm for matching shows how the join point in the base sequence diagram and pointcut matches. Hence using the matching semantics we can write the algorithm as

1. Matching (I, p, pcd)
2. If p ∈ I and p ∈ I. join point then pcd.kind ∈ join point
3. if p.type ∈ {message, lifeline} then pcd.name = I.name

In the first step, the algorithm takes the interaction (I), join point (p), and pointcut (pcd) as input. In the second step, the algorithm checks whether the join point belongs to the interaction. If the join point belongs to the interaction’s join point, then the kind of join point defined in a pointcut must belong to the join point. In the third step, the expression shows that if the types of join points are message and lifeline, then the name of the joint point defined in the pointcut must be matched with the name of the interaction’s join point.

#### Weaving algorithm

The weaving algorithm is used to weave the aspect into the base sequence diagram. This algorithm has two steps. The first step checks the matching process and has six sub-steps. If the matching process is successful, then step 1.1 takes the base sequence diagram I, its join point p, and aspect a with a pointcut pcd as inputs. Step 1.2 uses the add adaptation to add the security aspect. In step 1.3, the type of join point is checked, while step 1.4 checks whether the join joint belongs to the interaction. In steps 1.5 and 1.6, the algorithm selects the around adaptation position and substitutes the aspect with the join point.

1. *If* the matching process is successful *then*
  1.1 Input = (I, p, a.pcd)
  1.2 ad.kind = add
  1.3 p.type ∈ join point
  1.4 p *∈ I*. *join point*
  1.5 ad.position = around
  1.6 p’ = substitute ({p}, p → a)
2. *Else* weaving is *not* successful

### Verification of model

The verification of the resultant woven model in Fig 12 is performed in terms of correctness and completeness using theorems. The theorems prove that the resultant output model is complete and correct.

#### Verification of the woven model using the matching process

The verification of the woven model using the matching process is given below.

*Lemma 1*. *Correctness of the matching process*. Take a base sequence diagram I, a join point p, and the authentication pointcut pcd to check the correctness of the matching process. The expression (a) for the matching process can be written as

If Matching (I, p, pcd) where p ∈ I and p ∈ I.join point then I, p ⊢ match pcd.            (a)

We know that the join joint is the message, therefore the kind of pointcut and the type of join point is the message.

pcd.kind = message

p.type = message

Using the matching algorithm, the following Eq (b) can be written as

p.name = pcd.name                    (b)

p.name shows the name of the join point, while pcd.name shows the name defined in the pointcut. Using the message’s matching rules, we arrive at the following conclusion:

I, m ⊢ match pcd

The message m (Insert card) matches with the pointcut’s message m (Insert card). Thus, the matching is correct for the resultant woven model.

*Lemma 2*. *Completeness of the matching process*. Let a base sequence diagram I, a join point p, and a pointcut pcd specified in the mal sequence diagram in Fig 9. The completeness of the matching process depends on the correctness of the matching process. Therefore, the expression (a) can be written as

If I, p ⊢ match pcd then Matching (I, p, pcd).                (a)

In our case, the join joint = message, therefore using the matching algorithm

pcd.kind = message

pcd.name = m.name

p.type = message

We know that the type of join point p is a message m which belongs to an interaction I, therefore p ∈ I.joinpoint, there using the matching algorithm, we conclude that Matching = (I, p, pcd), Thus

If I, p ⊢ match pcd then Matching (I, p, pcd)

The expression (a) is proved. Therefore, the matching process for the aspect-oriented mal sequence woven model is correct and complete.

#### Verification of the woven model using the weaving process

The verification in terms of correctness and completeness of the weaving process is performed using mathematical theorems. The theorems are given below.

*Theorem 1*. *Correctness of the weaving process*. Take an interaction **I**, an aspect **a**, and a join point **p** to check the correctness of the weaving process performed for the woven model. Hence, we have to prove the following equation:

If Weaving (I, a, p) = I’’ then (I, a, p, weaving) → (I’’, a’, p’’, end)                    (a)

The induction has two cases, i.e., the base case and the induction hypothesis.

**1. Base case**

Using the base case for the weaving algorithm. We conclude

Weaving (I, a, p) = I

Using the weaving end rule, the equation becomes

(I, a, p, weaving) → (I, a, p, end)

**2. Induction hypothesis**

Let a = a’, we can write the Eq (a) as given below

If Weaving (I, a’, p) = I’’ then (I, a’, p, weaving) → (I’’, a’, p’’, end).                    (b)

We know that the matching process is correct and complete. The weaving process follows the below steps

1. Input = (I, p, a.pcd)
2. kind = add
3. type ∈ join point
4. p ∈ join point
5. position = around
6. p’ = substitute ({p}, p → a)

After performing the steps for weaving the models, the transformation equation can be written as given below.

(I, a, p, weaving) → (I’, a’, p’, weaving)                    (c)

Using hypothesis, we can conclude that

(I’, a’, p’, weaving) → (I’’, a’, p’’, end)                    (d)

Using the relation of transitivity for (c) and (d), the following Eq (e) becomes

**(I, a, p, weaving) → (I’’, a’, p’’, end)**                    (e)

From Eq (e), we can conclude that the models are correctly transformed into the woven model, which consists of interaction, a join point, and an aspect. Therefore, our woven model in Fig 12 is correct.

*Theorem 2*. *Completeness of the weaving process*. To check the completeness of the weaving process performed for the woven model. We have to prove the following Eq (a).

*If* Weaving (I, a, p, weaving) → (I’’, a’, p’’, end) *then* Weaving (I, a, p) = I’’            (a)

**1. Base case**

Using the weaving end rule, we conclude that

(I, a, p, weaving) → (I, a**’**, p, end)            (b)

Using the weaving algorithm, we can write the equation as

Weaving (I, a’, p) = I            (c)

**2. Induction hypothesis**

Let a = a’, we can write the equation as

*If* (I, a’, p, weaving) → (I’’, a’, p’’, end) *then* Weaving (I, a’, p) = I’’

The equation for before and after weaving is given below

Weaving (I, a, p) = Weaving (I’’, a’, p’’)            (d)

Since we know that Weaving (I’’, a’, p’’) = I’’,

Weaving (I, a, p) = I’’                    (e)

From this Eq (e) we can conclude that the woven model is complete. I’’ shows the output model in Fig 12. We conclude that the aspect-oriented mal sequence woven model has been successfully verified mathematically in terms of correctness and completeness. Hence, our null hypothesis is correct for RQ2.

## Research significance

The research is evaluated from different angles to determine whether the proposed approach addresses the basic security modeling problems and whether it provides significant benefits in terms of evolution, separation of concerns, and complexity reduction.

### Easier evolution

Using the research approach, the evolution of models is also easy without modifying the entire design. Both types of models, i.e., functional and security models, are separately managed and modified. If we want to modify the withdrawing cash model, we can change it separately from the security model.

### Better separation of concerns

The separation of security concerns is also improved using our approach. The authentication aspect is separated and localized. Similarly, we can model other security aspects and separately localize them.

### Reduced complexity

The effort and time needed for modification are also reduced which consequently reduces the complexity of models.

### Improved readability

The readability is improved as the base sequence model and security aspect model are less chaotic and easier to understand.

## Conclusion

Modeling software security threats using aspect-oriented techniques has received great attention from researchers in recent years. For this purpose, a lot of authors have modeled security using the UML but ignored security profiling using the UML extension mechanism in behavioral diagrams, specifically sequence diagrams. Moreover, the aspect-oriented profile is also ignored for security modeling. Hence, our contribution has made a clear difference to their work. Our first contribution is the modeling of authentication threats in the mal sequence diagram using the security profile and AOM profile. Our second contribution is the mathematical verification of the aspect-oriented mal sequence woven model in terms of correctness and completeness. Using the proposed approach, the scattering and tangling from the base sequence diagram have been successfully removed at the design stage using all the core concepts of the aspect-oriented technique. In the future, we want to determine the reduction rate in complexity, modeling effort, and time. We also want to model crosscutting constraints using aspect OCL for authentication threats using the proposed approach.

## Supporting information

S1 Dataset (DOCX)
