# Supplementary material for: Modeling and verification of authentication threats mitigation in aspect-oriented mal sequence woven model
Source: PLoS One. 2022 Jul 6;17(7):e0270702. doi: 10.1371/journal.pone.0270702 (PMC9258847; doi:10.1371/journal.pone.0270702)
Supplement: S1 Dataset — (DOCX) [file pone.0270702.s001.docx]

**Case Study – Dataset**

The functional and security requirements of automated teller machine (ATM) are given below.

1. **Functional Requirements:**

An ATM allows users to perform basic financial transactions such as

- Withdraw cash
- Bill payment
- Transfer cash
- Deposit cash
- Balance Inquiry
- Others

**1.1. User Requirements:**

The user requirements are given below

1. Select amount.
2. Select account type.
3. Withdraw amount.
4. Transfer amount
5. Deposit amount
6. Bill payment

**1.2. ATM Requirements:**

The ATM requirements are given below

1. Display the transaction options.
2. Displays the account balance.
3. Display cash options.
4. Deliver cash.
5. Request balance.
6. Eject card.
7. Scan card.
8. Print receipt.

**1.3. Card Scanner:**

The card scanner requirement is given below

1. Validate card

**1.4. ATM Card:**

The ATM card requirements are given below

1. Get PIN
2. Set PIN
3. Get account

**1.5. Bank:**

The bank requirements are given below

1. Verify account
2. Manage accounts
3. Save transaction details
4. Check the account balance

**1.6. Account:**

The account requirements are given below

1. Update account
2. Account type
3. Start transaction

**1.7. Transaction:**

The transaction requirements are given below

1. Get account balance
2. Process transaction
3. Complete transaction
4. Stop transaction
5. Cancel transaction
6. **Security Requirements:**

Based on the ATM card number, PIN, and fingerprint, the user is authenticated. For authentication, the requirements are given below.

1. Display a welcome message and prompt the user to enter an ATM card.
   1. The original card must be valid. The invalid card must be rejected.
2. Upon validation of the card, ATM asks the user to enter a four-digit PIN using the keypad.
   1. The PIN entered by a user must be matched with the account PIN. The Invalid PIN must be rejected.
   2. The attempts for the PIN must be less than 4.
3. Upon verification of the PIN, if the PIN is valid, the ATM requests a user’s fingerprint.
4. The ATM verifies the fingerprint, upon successful verification, the access is granted for the user to perform a transaction.
   1. The fingerprint entered by a user must be matched with the fingerprint. The Invalid fingerprint must be rejected.
   2. The fingerprint must be valid for Left Thumb while the session time must be equal to 15 seconds.
